# Bacterial Colonization and Tissue Compatibility of Denture Base Resins

**DOI:** 10.3390/dj6020020

**Published:** 2018-06-15

**Authors:** Constanze Olms, Maryam Yahiaoui-Doktor, Torsten W. Remmerbach, Catalina Suzana Stingu

**Affiliations:** 1 Department of Prosthodontics and Material Science, University of Leipzig, 04103 Leipzig, Germany; 2 Institute for Medical Informatics, Statistics and Epidemiology (IMISE), University of Leipzig, 04107 Leipzig, Germany; maryam.yahiaoui@imise.uni-leipzig.de; 3 Section of Oral Medicine, Department of Head Medicine and Oral Health, University of Leipzig, 04103 Leipzig, Germany; torsten.remmerbach@uniklinik-leipzig.de; 4 Institute for Medical Microbiology and Epidemiology of Infectious Diseases, University Hospital of Leipzig, 04103 Leipzig, Germany; CatalinaSuzana.Stingu@medizin.uni-leipzig.de

**Keywords:** bacteria, polymethyl methacrylate, polyamide, cell biology

## Abstract

Currently, there is minimal clinical data regarding biofilm composition on the surface of denture bases and the clinical tissue compatibility. Therefore, the aim of this experimental study was to compare the bacterial colonization and the tissue compatibility of a hypoallergenic polyamide with a frequently used PMMA resin tested intraorally in a randomized split-mouth design. Test specimens made of polyamide (*n* = 10) and PMMA (*n* = 10) were attached over a molar band appliance in oral cavity of 10 subjects. A cytological smear test was done from palatal mucosa at baseline and after four weeks. The monolayers were inspected for micronuclei. After four weeks in situ, the appliance was removed. The test specimens were immediately cultivated on non-selective and selective nutrient media. All growing colonies were identified using VITEK-MS. The anonymized results were analyzed descriptively. A total of 110 different bacterial species could be isolated, including putative pathogens. An average of 17.8 different bacterial species grew on the PMMA specimens, and 17.3 on the polyamide specimens. The highest number of different bacterial species was *n* = 24, found on a PMMA specimen. On the two specimens, a similar bacterial distribution was observed. Micronuclei, as a marker for genotoxic potential of dental materials, were not detected. This study indicates that the composition of bacterial biofilm developed on these resins after four weeks is not influenced by the type of resin itself. The two materials showed no cytological differences. This investigation suggests that polyamide and PMMA are suitable for clinical use as denture base material.

## 1. Introduction

The oral microbiome consists of viruses, bacteria, fungi, and protozoa. The microorganisms colonize different habitats in the oral cavity, such as the dorsum of the tongue, the tongue margin, the hard and soft palate, the cheek, the vestibule, the tonsils and the supra- and subgingival biofilm. The bacteria live in close proximity within the biofilm, which has different levels of complexity in each individual [1,2]. Dental plaque has a diverse microbial composition, with many species being detected at individual sites.

Specified areas between the basal and oral surfaces of a mucosa-supported prosthesis could be found in patients [3]. Acidic conditions prevail in the plaque on the side of the palate, where the growth of *Streptococci* and *Candida* spp. is favored [3]. The prosthetic plaque may include Gram-positive rods, such as *Actinomyces israelii*, and Gram-negative rods in small numbers. *Staphylococcus aureus* has also been isolated from prosthetic plaques [4]. Dentures of elderly are often contaminated with pathogenic bacteria and can affect the general health status [5].

Inadequately cleaned dentures rapidly form a biofilm on their surface [6]. An anaerobic atmosphere which is, for instance, found under poorly cleaned denture bases, promotes the proliferation of certain bacterial species, and may lead to a pathogenic biofilm composition [2]. This can lead to altered pH levels, and trigger inflammatory processes in the adjacent oral tissues [7].

Factors that have a decisive influence on this process are the fit of the prosthesis, the increasing age of the prosthesis wearer, and the colonization of bacteria and fungi with poor prosthetic care [8,9,10]. Reduced salivation or xerostomia can also affect the adherence of microorganisms. The study by Hahnel et al. (2014) examined 68 elderly patients (>60 years), of whom 16% had a xerostomia, and 31% had a reduced salivary flow rate (hyposalivation) [11]. Insufficiently cleaned prostheses rapidly form a biofilm on their surface [6], and may pose a general health risk [12]. A recent study by the University of Glasgow’s Dental Hospital and School (UK) showed that two-thirds of poorly-cleaned prostheses were burdened with pulmonary-pathogenic bacteria [5]. About 37% of patients had prosthetic stomatitis. The pathogenic bacteria *Pseudomonas aeruginosa*, *Streptococcus pneumoniae*, *Haemophilus influenzae B*, *Streptococcus pyogenes*, and *Moraxella catarrhalis* have been shown to cause severe respiratory disease. The prevalence of the pathogens did not differ significantly in healthy and inflamed mucosa.

The biomass of the microbial biofilm consists of about 80% bacteria. In the literature, only the intraoral surfaces are described in vivo for bacteria adhesion. For this reason, it is also important to analyze the bacteria growth on the basal side from denture base resins under standardized conditions. In the literature the period of investigation was extended from 1 to 14 days [13,14,15,16]. Samples of microbial plaques of longer growth periods (weeks, months, years) were taken directly from the current dental prosthesis [17], and did not correspond to the criteria of a standardized procedure.

For a standardized procedure, the abovementioned factors for a pathogen biofilm (age, xerostomia, gingivitis, and periodontitis etc.) under denture base resins have to be disabled. For this reason, it is necessary to determine the individual bacteria growth in an experimental study with younger subjects. The study of Bourgeois et al. showed that only 28% of 20–35 years old caries-free (DMFT = 0) and oral healthy subjects (*n* = 25) were carrying *Candida albicans* in their interdental biofilms [18]. These studies assumed a direct correlation between dental caries bacteria and *Candida albicans* [19,20]. The mean number of *Candida* increases with age [18,21].

Further, dental materials have an influence on the formation of micronuclei [22,23,24,25], an indicator of genotoxic exposure. Currently, there is minimal clinical data regarding biofilm composition on the surface of denture bases, and the clinical tissue compatibility. It is known that subjects with periodontal diseases showed a higher micronuclei rate. Missing teeth and removable dentures also play a role in the biofilm composition [22].

Conventional dental materials, like polymethylmethacrylate (PMMA) can be associated with adverse reactions of the oral mucosa [26,27,28,29]. Alternative denture base materials, such as polyamide, were developed. These alternative or hypoallergenic materials have frequently been compared to PMMA resins concerning physical–mechanical properties, but the clinical compatibility of these materials has hardly been investigated. Both materials are used for partial and full denture base in direct contact with the mucosa.

Therefore, the aim of this experimental study was to compare the bacterial colonization and the tissue compatibility of a hypoallergenic polyamide with a frequently used PMMA resin tested intraorally in a randomized split-mouth design in young adults.

## 2. Materials and Methods

The Ethical Committee of the Medical Faculty, University of Leipzig, Germany approved the protocol, including clinical measurements and the sampling procedure (030-14-27012014). The principles outlined in the Declaration of Helsinki, in its latest version from the 64th WMA general meeting in October 2013 in Fortaleza (Brazil), were followed (World Medical Association 2013). All subjects were informed of the nature, potential risks, and benefits of participation in the study, and signed an informed consent prior to enter into the study (Table 1).

**Figure 1:**
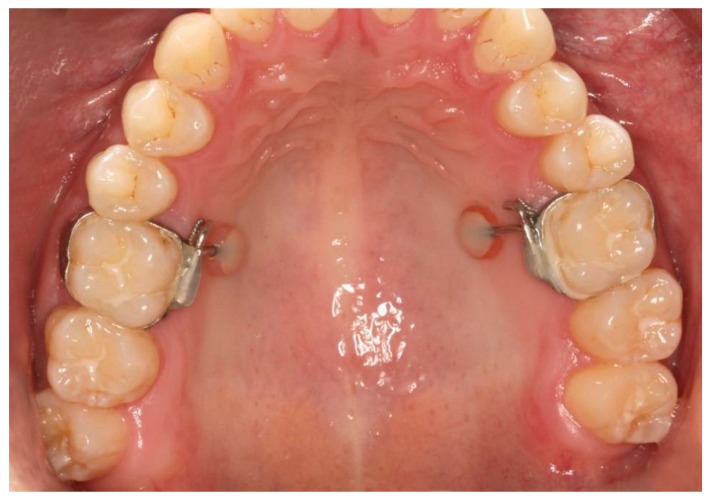
Intraoral test specimen after four weeks.

A hypoallergenic polyamide (Valplast, Valplast Inc., Westbury, NY, USA) and a conventional PMMA resin (PalaXPress, Heraeus Kulzer GmbH, Hanau, Germany) were applied intraorally (diameter 5 mm) using a molar band appliance (Figure 1).

The two test specimens were prepared differently. For the preparation of the polyamide test specimens, prefabricated platelets from the manufacturer were used, which were ground with a size of 5 mm diameter and 2 mm thickness.

The PMMA specimens were produced in accordance with manufacturers’ guidelines. A mold with a silicone impression material (VPS Hydro Putty Soft, Henry Schein Europe, Langen, Germany) provided as a negative form. After removal, the produced PMMA specimen was also ground to a size of 5 mm in diameter and 2 mm in thickness.

The surfaces of both materials were polished in accordance with manufacturers’ guidelines. They were left in situ four weeks. After fitting the appliance, visits took place every seven days. The participants removed any food residues daily using dental floss. In the area of the test specimens, the occurrence of inflammatory signs, such as swelling, redness, and pain, was elevated to the control dates. At the time of insertion, all intended mucosal areas were clear of signs of inflammation, and the mucosa was pale pink and moistened.

**Figure 2:**
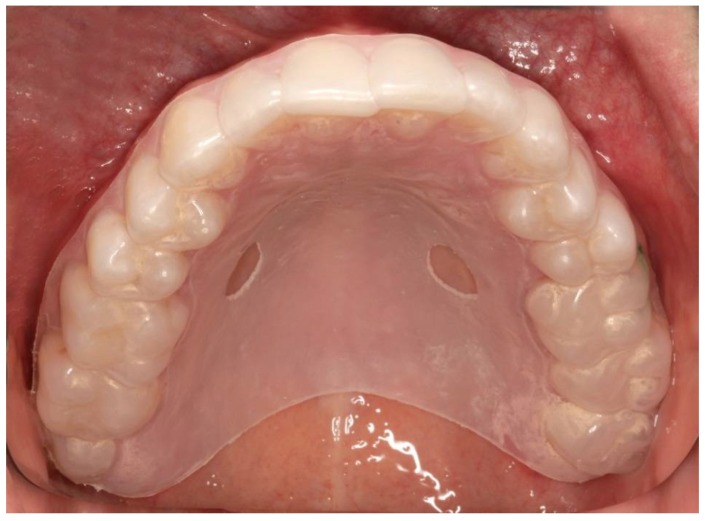
Individual splint for mucosal smear.

An individual splint was required for each test person to precisely locate the position during the sampling and to ensure the same smear areal. All splints were produced with a thermoforming machine (Vacfomat U, Dreve Dentamid GmbH, Unna, Germany) according to the manufacturer’s guidelines. The splint was inserted into the upper jaw of the test person to sign the exact position of the test specimens. The notch was chosen according to the test specimens. Afterwards the smear sampling was carried out with the aid of this fenestrated splint, before and after insertion of the appliance (Figure 2).

After the fourth follow-up examination (four weeks in situ), the appliance was removed.

## 3. Microbiological Assessments

Immediately after removal, the area of the specimens which were not relevant for the microbial sample collection was cleaned with 96% alcohol (Th. Geyer GmbH & Co. KG, Renningen, Germany). Accordingly, the test specimens were placed in an Eppendorf tube containing BHI (brain heart infusion) broth (Oxoid, Basingstoke, UK), and packed immediately in a GENbag Anaer (Biomerieux, Lyon, France). The specimens were transported within 20 min to the Institute of Medical Microbiology and Epidemiology of Infectious Diseases, Leipzig. The samples were then vortexed with glass beads for 30 s. BHI dilutions (10^−1^ to 10^−5^) of all the samples were cultured on blood agar (Oxoid, Basingstoke, UK) and Columbia blood agar plates (Oxoid, Basingstoke, UK) supplemented with 5% sheep blood, hemin (5 µg/L) (Sigma, Taufkirchen, Germany), and vitamin K1 (1 µg/L) (Sigma, Taufkirchen, Germany). The blood agar plates were incubated aerobically at 37 °C for two days. The Columbia plates were incubated anaerobically (Whitley MG 1000, anaerobic workstation, (Meintrup Laborgeräte GmbH, Lähden-Holte, Germany) at 37 °C for seven days. All different colonies were counted (results were expressed as cfu/mL), and were further subcultivated and then identified by VITEK-MS (bioMerieux, Lyon, France), using the commercially V2.0 knowledge database for clinical use. The colonies which were not identified by VITEK-MS were subjected to sequence analysis of the 16S rRNA gene. The strain sequences were compared with sequences deposited in GenBank using the program BLAST through the NCBI server, and with the sequences deposited in the Human Oral Microbiome Database (HOMD) using the program HOMD 16S rRNA Sequence Identification [30].

## 4. Cytology Assessments

The cell collection method was carried out using Orcellex Brushes (Rovers Medical Devices B.V., Oss, The Netherlands) according to the procedure described above.

Two brushes per sampling site were used to obtain a representative amount of epithelial cells from palatal mucosa before intervention (baseline T1) and after four weeks (T2). Following the brush biopsies by Orcellex Brushes, fixation was carried out by separating the brush head from the applicator and placing it into the fixation liquid in the BD SurePath Collection Vial (BD Diagnostics—TriPath, Erembodegem, Belgium). In the laboratory of the Institute of Pathology (Mathias-Hospital, Rheine, Germany) the preparation of thin layers (or monolayers) was done by the preparation procedure for SurePath preparations, which was originally developed for cervical smears [31]. Thin layers, with a so-called settling chamber with a diameter of 13 mm, were produced according to the manufacturer’s guidelines. The automatic staining of the preparations was carried out using the staining solutions according to the manufacturer guidelines. After staining, the glass slides were covered and stored at room temperature until evaluation. All monolayers were screened systematically and completely in the longitudinal direction of the slide. The evaluation of 100 epithelial cells per preparation was carried out, whereby specifically those epithelial cells were selected that were not superimposed with other cells or with themselves. The cells were evaluated for their cell shape, and for the presence and appearance of the nucleus.

In order to compare the two denture base resins, the micronucleus test was used for cytological preparation and microscopic examination. Micronuclei (MC) are described as round or oval visible light microscopic chromatin components located near the main nucleus [32,33]. They are small additional nuclei in the cytoplasm and can be seen microscopically as extranuclear DNA material [34,35,36]. MC are increasingly produced in the presence of genotoxic substances, either as a result of dysfunction of the spindle apparatus or as a result of chromosome breakage [32,36,37].

The study was done with a reduced number of probands because the feasibility of the method standing in the foreground.

The anonymized results were descriptively analyzed with SPSS 23.0 (SPSS Inc., Chicago, IL, USA).

## 5. Results

At the time of removal, a subtle impression with a very slight red spot (<1 mm) was observed on one polyamide and one PMMA test specimen (different subjects). In both cases there was an increased pressure of the specimen on the underlying tissue, due to a tongue press on the palate.

### 5.1. Microbiological Results

One hundred and ten different bacterial species were found in total on the 20 specimens from 10 subjects. An average of 17.5 different bacterial species was isolated per subject, 17.8 different bacterial species grew on the PMMA specimens, and 17.3 on the polyamide specimens. Increased growth of anaerobic bacteria was found on the test specimens with the higher number of species, regardless of the material type. The highest number of different bacterial species was *n* = 24, found on a PMMA specimen.

A similar distribution was observed on both specimens. Seven bacterial species could be detected in more than 50% of the specimens, and included the genera *Actinomyces*, *Atopobium*, *Capnocythophaga*, *Neisseria*, *Prevotella*, and *Streptococcus* (Table 2). *Veillonella parvula* could be isolated in all cases. *Staphylococcus aureus* was not detected on any of the samples. In two subjects, *Staphylococcus epidermidis* was detected.

### 5.2. Cytology Results

A total of 40 glass slides were included in the evaluation. They showed a uniform distribution of the cells in the circular evaluation area. In addition, superposition of the cells with other cells or with themselves occurred very rarely. There was hardly any foreign material. As described, MC testing was performed on all thin layers. All cells were carefully screened for extranuclear material with the typical characteristics of MC, but no MC were found.

The following table gives an overview of the results at the different control times of the study (Table 3).

The oral mucosal smear taken before insertion of the molar bands (T1) with the specimen attached had many cells with no, or very small, pyknotic, intensely stained nuclei, and with much cytoplasm. Keratinized cells predominate, forming the superficial cell layer [38].

After the four-week period of wear (T2) of the apparatus, more cells with a clear cell nucleus were present. These probably came mainly from the intermediate cell layer [38]. Furthermore, an increased occurrence of cell clumping and assembly of cells was detected.

## 6. Discussion

This clinical split-mouth study investigated a PMMA denture resin and a methacrylate-free polyamide regarding their microbial colonization over a period of four weeks. Both materials showed a similar bacterial growth and did not seem to be influenced by the nature of the denture basis material. MC as a marker for genotoxic potential of dental materials were not detected.

First, some aspects of the materials and methods used will be discussed. A pilot study was conducted, which examined the feasibility of the method in the foreground, whereby the investigation was carried out on a reduced number of cases (*n* = 10). Recruitment considers a number of inclusion and exclusion criteria. Prerequisite was to obtain a homogeneous group of subjects, where good compliance and good oral hygiene were crucial.

The use of the molar ligaments is a new method for clinical biocompatibility testing of dental materials. A comparable study could not be found in the current literature. The main advantage to using a fixed model is to extend the wearing time. A not yet published investigation showed that this intraoral testing method have not influenced the oral health-related quality of life of a study group (*n* = 10) compared with a control group (*n* = 10) during four weeks of wearing [39]. No functional impairment was observed. The test specimens were placed on with basal side pressureless over the mentioned examination period of the mucosa.

In contrast to the investigations of biofilms on material surfaces [40], a qualitative and quantitative determination of the bacteria was carried out in the present study. Samples for microbiological examination were taken directly from the basal surface. The area of the specimens, which were not relevant for the microbial sample collection, was cleaned with 96% alcohol. The Microbiology Institute was located only 10 min away from the dental clinic, so all samples could be further processed less than 20 min after removal.

Since it is well known that conventional biochemical tests may fail to discern some of the newly described oral taxa, the recently established MALDI-TOF-MS methodology was used in this study. Nevertheless, VITEK-MS using the commercial V2.0 knowledge base for clinical use database was not able to identify all the isolated bacteria, so they were subjected to sequence analysis of the 16S rRNA gene.

Liquid-based cytology was chosen, whose advantages are described in detail by Olms et al. [41]. In order to obtain sufficient cell material, two brushes per sampling site were used [31]. The procedure of the liquid-based cytology also resulted in a much more even distribution of the cells on the slide, and thus, less cell overlapping [42,43,44,45]. Another important advantage of this method was that the unwanted harvested material, such as blood cells, debris, and mucous, was almost completely filtered out before being transferred to the slides [43,44,45]. Therefore, the preparations showed less impurities, and the background appeared clearer [42].

Under the present experimental conditions, a stable biofilm had developed over four weeks on the basal areas of the specimens, and it corresponds to a typical colonization [3]. If the plaque accumulates undisturbed, the proportion of bacteria in the biofilm shifts [3]. The metabolism of the pioneer species (aerobic, facultative aerobic) consumes the oxygen. Carbon dioxide or other gases are formed as the end product of the microbial metabolism. The pH decreases, and thereby promotes the growth of obligate anaerobic species. An anaerobic atmosphere, e.g., under poorly cleaned denture bases, may favor the propagation of certain bacterial species, and may lead to a pathogenic composition of the biofilm [2,46]. Thus, the analysis of the present plaque samples showed a composition similar to that of a prosthetic plaque [3].

A total of one hundred and ten different bacterial species, including putative periodontitis and endocarditis associated pathogens, were found on the 20 specimens. Microbiological examination showed that there were no quantitative or qualitative differences in biofilm growth on the two test specimens.

In the present study, no *Candida albicans* strains were found on tested specimens. The study of Bourgeois et al. showed that only 28% of 20–35 year old caries-free (DMFT = 0) and oral healthy subjects (*n* = 25) were carrying *Candida albicans* in their interdental biofilms [18]. Studies found a direct correlation for dental caries bacteria and *Candida albicans* [19,20]. The mean number of *Candida* increases with age [18,21]. The study of Moalic et al. showed that the presence of *Candida albicans* depended on the gender of the subjects (*n* = 353, average age 21.3 years). The fungal colonization with *Candida albicans* was statistically more frequent in males than in females [20]. This result was not observed in our investigation (male *n* = 7; female *n* = 3). Our participants had a very good oral hygiene, were free from caries and lesions, and no showed signs of gingivitis or periodontitis. It is stated in the literature that prosthetic base materials can affect the attachment and propagation of *Candida albicans* [47,48]. Thus, the residual monomer content in the PMMA can lead to reduced adhesion and growth inhibition of *Candida* [49,50]. The in vitro study by Susewind et al. (2015) investigated the growth of *Candida albicans* on four different denture base materials over a period of 188 h. The lowest growth rate was found on the MMA, and the highest on the metallic surface [51]. The study by Freitas Fernandes et al. (2011) showed a significantly higher growth rate of *Candida* biofilm on a polyamide compared to a PMMA [52].

There were no cytological differences between the smears of the two tested specimens that could mean that both the smears of the respective half of the jaw with PMMA and the smears of the respective jaw half with polyamide provided the same cytological results regarding the morphology of the cell nuclei and cell forms.

One possible cause that MC were not found is that the MC test was not the appropriate test for this study. This may be because of the damage to the DNA was not measurable, because the test was not sensitive enough. In addition, it is possible that the selected contact time of the tested specimens with the palate mucosa of subjects was too short. Duration was limited to four weeks in this study. Furthermore, the choice of the time window of four weeks is limited to at least one cell cycle. In addition, it may be that the absence of MC is an “aging phenomenon”. Since the age of the subjects in this study is between 20 and 30 years, it is possible that the study participants for the occurrence of MC are too young. Comparing the average subject age with the age of study participants, the subjects are between 30 and 60 years old, and the mean age was 42.4 years [22]. In this study, the average age was only 24.0 years. The young age, the absence of medication, no tobacco or alcohol consumption, and the good hygiene of the subjects can, therefore, explain the absence of MC. This is different to the study of Bloching et al. [22].

Further clinical investigations which should include elderly adults with removable dentures are necessary for assessment of clinical relevance.

Consequently, the basal surfaces of dentures are always rougher than the oral surfaces. Whether denture saddles with a highly polished base lead to a reduction in microbial flora must be investigated in further clinical studies.

Further in vitro studies are needed to monitor changes of the bacterial composition on the surface of such resin specimens regarding their capacity of survival and interaction with other bacterial species after longer periods of time.

## 7. Conclusions

Polyamides, as methacrylate-free resins compared to conventional PMMAs, showed no difference in microbial colonization. Within the limits of this study, it is suggested that the composition of bacterial biofilm developed on these resins after four weeks is not influenced by the type of resin itself. After four weeks the polyamide and PMMA showed no cytological differences. No micronuclei were detected in this period of investigation. Polyamide and PMMA are suitable for clinical use as denture base resins. Further investigation in form of a larger prospective clinical study, also including older adults, is necessary.

## Figures and Tables

**Table 1:** Inclusion and exclusion criteria.

Inclusion Criteria	Exclusion Criteria
Age between 20 and 30 years	Smoker
Healthy	Pregnancy
No signs of gingivitis or periodontitis	Edentulousness
No caries lesions	Fixed partial dentures
Fully dentate	Removable partial dentures
good mouth hygiene	Insufficient fillings
good compliance	Composite fillings more than 5 per person and more than ten years old
	Amalgam fillings
	Known intolerance or allergies to the materials to be used
	Oral mucosal diseases (e.g., lichen planus, leukoplakia)
	Systemic diseases (e.g., diabetes mellitus)
	Taking immunosuppressants
	Antibiotic therapy in the last 6 weeks
	Non-consent by the subject

**Table 2:** Occurrence of most frequent isolated bacteria.

Proband	1 (m)	2 (m)	3 (f)	4 (f)	5 (f)	6 (m)	7 (m)	8 (m)	9 (m)	10 (m)
**Number of different isolated bacteria per sample**										
PMMA	18	15	20	16	24	18	22	11	16	18
Polyamide	19	9	15	19	18	18	18	14	21	22
**Most frequent isolated bacteria (cfu/mL)**										
PMMA										
*Actinomyces naeslundii*	-	1,000,000	2,000,000	100,000	-	200,000	1,010,000	100,000	-	100,000
*Actinomyces odontolyticus*	120,000	1,210,000	1,000,000	1,300,000	4,000,000	-	-	340,000	-	500,000
*Atopobium parvulum*	10,000	100,000	1,000,000	1,000,000	2,000,000	1,000,000	-	100,000	200,000	1,000,000
*Capnocythophaga gingivalis*	1,020,000	100,000	-	200,000	1,000,000	-	-	220,000	100,000	-
*Neisseria mucosa*	-	-	-	100,000	1,000,000	100,000	10,000	100,000	200,000	10,000
*Prevotella melaninogenica*	10,000	200,000	1,000,000	400,000	-	1,110,000	-	-	-	100,000
*Streptococcus gordonii*	100,000	-	-	-	100,000	-	-	10,000	410,000	2,000,000
*Streptococcus mitis Gruppe*	-	1,210,000	3,100,000	-	1,000,000	1,300,000	10,000	100,000	1,300,000	-
*Streptococcus parasanguinis*	-	10,000	-	200,000	-	100,000	20,000	-	-	1,700,000
*Streptococcus sanguinis*	-	-	-	100,000	2,000,000	-	100,000	400,000	500,000	100,000
*Veillonella parvula*	1,110,000	1,400,000	-	2,000,000	2,000,000	110,000	1,100,000	300,000	200,000	1,300,000
Polyamide										
*Actinomyces naeslundii*	1,100,000	10,000	1,100,000	1,500,000	-	1,210,000	1,330,000	-	-	-
*Actinomyces odontolyticus*	1,200,000	-	1,100,000	2,300,000	1,000,000	-	200,000	400,000	100,000	1,100,000
*Atopobium parvulum*	1,000,000	-	-	-	-	-	-	200,000	100,000	1,000,000
*Capnocythophaga gingivalis*	-	-	-	-	-	-	-	-	100,000	-
*Neisseria mucosa*	1,000,000	-	-	-	10,000	1,100,000	10,000	10,000	-	-
*Prevotella melaninogenica*	-	-	-	100,000	-	-	-	100,000	-	1,000,000
*Streptococcus gordonii*	1,110,000	-	-	100,000	-	-	-	700,000	100,000	-
*Streptococcus mitis Gruppe*	1,100,000	-	-	-	10,000	1,200,000	100,000	200,000	2,200,000	-
*Streptococcus parasanguinis*	210,000	-	100,000	-	1000,000	1,100,000	-	-	1,000,000	100,000
*Streptococcus sanguinis*	-	-	1,000,000	300,000	1,110,000	-	110,000	100,000	1,000,000	-
*Veillonella parvula*	1,000,000	100,000	1,000,000	-	1,100,000	1,310,000	1,110,000	100,000	300,000	2,000,000

Legend: cfu—colony forming units, m—male, f—female.

**Table 3:** Results of cytology.

	PMMA			Polyamide		
	Average Number of Cells per 100 Evaluated Cells			Average Number of Cells per 100 Evaluated Cells		
	Cells with Nucleus	Cells without Nucleus	MC	Cells with Nucleus	Cells without Nucleus	MC
T1-I. Quadrant	10	90	0	7	93	0
T1-II. Quadrant	8	92	0	9	91	0
T1-total	9	91	0	8	92	0
T2-I. Quadrant	30	70	0	36	64	0
T2-II. Quadrant	34	66	0	34	66	0
T2-total	32	68	0	35	65	0

Legend: T1—baseline, T2—after four weeks, MC—micronuclei.
